# Survey of husbandry practices and captive environments for North Island brown kiwi *(Apteryx mantelli)* housed in facilities within and outside New Zealand

**DOI:** 10.1017/S0962728625100109

**Published:** 2025-06-20

**Authors:** Rebecca L Connor, Nicholas Ling, Ngaio J Beausoleil, Kris Descovich, Todd Jenkinson

**Affiliations:** 1 Te Aka Mātuatua School of Science, University of Waikato, New Zealand; 2 Animal Welfare Science and Bioethics Centre, School of Veterinary Science, Massey University, New Zealand; 3 School of Veterinary Science, The University of Queensland, Australia; 4 Zoo and Aquarium Association, New Zealand

**Keywords:** animal welfare, captive management, husbandry, kiwi, nocturnal house, zoo

## Abstract

North Island brown kiwi (*Apteryx mantelli*), endemic to New Zealand (NZ), are held in captivity both within and outside of NZ. However, more knowledge is required regarding how kiwi are housed and managed. This study aimed to characterise the demographics and reported health/behavioural issues of the captive population of kiwi, investigate current housing and husbandry practices, and explore the association between reported behavioural problems and housing practices. Between November 2021 and June 2022, all 31 facilities holding kiwi were invited to participate in a questionnaire. Thirteen facilities within NZ (92.9% response rate) and ten elsewhere in the world (58.8%) responded, covering 97 kiwi in NZ (93.3%) and 40 outside NZ (83.3%). Kiwi in NZ were younger on average than birds elsewhere. Environmental conditions, including enclosure size, temperature, and lighting, varied across facilities. Health issues were reported in 39% of kiwi and behavioural in 20%, with common behavioural issues including stereotypical or reproduction-related behaviours. Kiwi in those facilities outside of NZ were heavier and housed in smaller enclosures. Kiwi in nocturnal houses were more likely to be reported as displaying behavioural problems than off-display enclosures. A higher proportion of NZ kiwi were housed in nocturnal houses compared to elsewhere, and one in five NZ kiwi were reported as displaying a behavioural problem, compared to 1/8 in other countries. Behavioural issues in kiwi may be underreported due to their nocturnal nature, and both behavioural and health challenges could negatively impact their welfare. Further research is essential to optimise captive conditions and improve health, behaviour, and welfare outcomes for this iconic species.

## Introduction

New Zealand (NZ) is home to the iconic and endemic kiwi, of which there are five species. The North Island brown kiwi (*Apteryx mantelli*) is the most common, accounting for approximately 28,000 of an estimated 68,000 kiwi (New Zealand Department of Conservation n.d.). Within the species, four distinct geographic subgroups are recognised: the Northland, Coromandel, eastern and western populations (Weir *et al.* 2016). North Island brown (NIB) kiwi are classified as not threatened (conservation dependent) by the NZ Department of Conservation. However unmanaged wild populations are declining at a rate of 2% per year, mainly due to predation from introduced mammals, such as stoats (*Mustela erminea*), ferrets (*Mustela furo*), dogs (*Canis lupus familiaris*), and cats (*Felis catus*) (Burns 2014; Fraser & Johnson 2015). Human activities, including land clearing, vehicles, and pest management have also impacted kiwi numbers and geographical distribution (Germano *et al.* 2018).

NIB kiwi are held in captive facilities both in NZ and overseas for conservation education/advocacy and breeding. In NZ facilities kiwi are held under permits granted by the NZ Department of Conservation and are further supervised and managed by the regional accrediting body, the Zoo and Aquarium Association Australasia (ZAA). At the time of writing, the only facility not currently accredited by ZAA were applying for accreditation. Regardless, they were still required to adhere to the recommendations of the programme as regards to breeding/transfers/release. As NZ captive facilities currently have only limited space, kiwi management is concentrated on only one taxon (species and geographic provenance) (Barlow 2011; Germano *et al.* 2018). The eastern NIB kiwi was selected because it was already well represented in captivity, including a number of individuals brought in from the wild that were unrelated to all others in the population, and because existing wild populations were anticipated to benefit from supplementation (Barlow 2011). The occasional kiwi of other species still lives in captivity as a result of failed rehabilitation/physical impairment.

In contrast, the kiwi population kept outside of NZ is made up of predominantly Northland and mixed-region NIB kiwi and birds were last exported in 2010 to increase the genetic diversity of this population (Barlow 2011; 2018). This overseas population is jointly managed through the Species Survival Programme (SSP) in the USA and the European Endangered Species Programme (EEP) (Barlow 2011). The ZAA and the joint SSP/EEP are overseen by studbook keepers who decide on breeding recommendations and transfers between facilities. While information is informally shared between programmes, no overarching standards exist for kiwi management, and neither ZAA nor the NZ Department of Conservation has jurisdiction over kiwi once they have left NZ.

Current recommendations for the captive management of NIB kiwi are outlined in the Brown *Kiwi (*Apteryx mantelli*) Husbandry Manual* (Fraser & Johnson 2015), hereafter the ‘*Manual*’. The *Manual* was developed in 2011 and updated in 2015, using the collective knowledge of keepers and managers around NZ and based on the established science at the time, as well as anecdotal evidence and keeper experience (Fraser & Johnson 2015). The *Manual* outlines minimum standards and recommended best practices for many areas of husbandry, however, it lacks specific requirements for others that may be important for kiwi management or welfare. For example, lighting levels are recommended as being bright enough “for visitors to see the kiwi clearly while still being dark enough to encourage the birds to forage in the enclosure” (Fraser & Johnson 2015; p 23). However, there are no standards or recommendations for the lux level or light spectrum and the effects of these and other environmental parameters on kiwi health, behaviour, and welfare are unknown. Therefore, for some aspects of housing and management, facilities rely upon non-specific recommendations or those lacking scientific foundations.

Kiwi present particular challenges for captive management, firstly because they are nocturnal and secondly because many are being prepared for breeding and/or release into the wild, which means they need to retain physiological and anatomical health, especially reproductive function, and appropriate behaviour (Crates et al. 2023; Teixeira *et* *al.* 2007). To facilitate public viewing, kiwi are often displayed in a nocturnal house; an indoor enclosure where the day/night cycle is reversed. Nocturnal houses are used worldwide to display nocturnal animals, but while there is acknowledgement of the difficulties presented by housing captive animals in nocturnal houses, such as the effect of the colour of artificial lighting (Fuller *et al.* 2016), or of enhanced olfactory senses, visitor movement and camera flashes, or sensitivity to vibrations or elector-magnetic fields (French *et* *al.* 2024) and the need for observations to occur both 24 h a day, and over all seasons (Brando & Buchanan-Smith 2018), there is still little research investigating the welfare of animals within these enclosure types.

While efforts are made to replicate the wild environment in nocturnal houses, there are limitations. The lighting, soundscape, food availability and presentation, humidity and temperature are all necessarily artificially controlled within ranges recommended in the *Manual*, based on the best current knowledge. However, as noted above, whether the recommended ranges are optimal is not well understood.

While the general impacts of captivity are well documented (Clubb & Mason 2003; Jakob-Hoff *et* *al.* 2019; Mason 2010), little is known about the effects of captive management on kiwi behaviour and welfare, with only two studies having been published. In one, two juvenile kiwi (8 to 11 months) in a US facility were found to be most active between 1800 and 2200h and to spend more time preening after midnight (Wesley & Brader 2014). Individual variation was observed in locomotive behaviour, such as stereotypical pacing, probing/walking, and running. Likewise, behaviour was found to vary significantly among individual birds in a NZ study of 15 kiwi in four facilities (Davison *et al.* 2021). Birds responded to loud visitor and environmental noise with pacing and startle responses. However, with little understanding of the wild behaviour of kiwi, it is difficult to determine ‘normal’ from ‘abnormal’ behaviour for captive animals to evaluate impacts on their welfare. Further research shows that captive chicks grow at a faster rate than their wild counterparts while having shorter bills at birth, an issue that may be related to the captive diet (Prier *et al.* 2013). In addition, testosterone levels were higher during incubation in captive males than in wild males, which could cause lower sperm levels and contribute to low captive fertility (Jensen *et al.* 2019).

NZ facilities holding kiwi are evaluated based on animal welfare by the ZAA. In order to achieve accreditation, facilities must provide evidence that the welfare needs of animals are being met in terms of nutrition, environment, physical health and behavioural interactions (Zoo and Aquarium Association of Australasia 2024). However, these records are not publicly available, and it is otherwise largely unknown how NZ and overseas facilities manage kiwi, the environmental conditions they provide, and how their management practices relate to the *Manual* recommendations. Nor is it known what effects existing management practices and environment might have on kiwi health, behaviour and overall welfare. Therefore, the aims of this study were to characterise aspects of the captive population of kiwi both within and outside New Zealand, including demographics and reported health and behavioural issues, investigate current housing and husbandry practices, and explore associations between reported behavioural problems and enclosure type. These data will highlight key research priorities for the future.

## Materials and methods

Data on kiwi housing, husbandry, health and behaviour were gathered using a questionnaire distributed to all facilities holding NIB kiwi in October 2021. The questionnaire was developed following informal conversations with key NZ stakeholders (zoo managers, keepers, ZAA staff, and scientists) and the ZAA kiwi studbook manager to determine gaps in current knowledge and features of kiwi housing and husbandry believed to vary among facilities. These conversations took place during initial site visits to various facilities and during the NZ National Kiwi Hui 2021, a conference aimed at those involved in captive and wild kiwi management.

Facilities were invited to participate via email, after which a Zoom call was arranged to explain the use of logic (i.e. if ‘no’ was selected then the survey would skip to the following section) and the various sections of the questionnaire. Facilities then had two weeks to complete the questionnaire, after which a follow-up Zoom call was held to clarify any information.

The questionnaire was developed and distributed in English using the Qualtrics platform (Qualtrics, Provo, UT, USA). It took between 15 min and 1 h to complete, depending on the number of enclosures and kiwi held by the participating facility (see Questionnaire development and structure, Appendix A; Supplementary material). The questions were divided into sections based on different features of kiwi housing and husbandry (see Full questionnaire, Appendix B; Supplementary material). All research procedures were approved by the University of Waikato Human Research Ethics Committee (approval HREC[HECS]2021#53).

## Data treatment and analysis

Data were exported from Qualtrics into Microsoft Excel® (Microsoft Corporation® 2018). Prior to analysis, numerical data from open-ended questions regarding enclosure size, kiwi age and weight were converted to the same units: kiwi ages were converted into months, weight to grams and enclosure size to m^2^. Kiwi were categorised into age groups according to the *Manual* (Robertson & Colbourne 2017): Chick (0 to 10–50 days), juvenile (10–50 days to 6 months), subadult (6 months to 4 years) or adult (over 4 years or when breeding begins). When not provided directly, the area of each enclosure was calculated by adding the area of indoor and outdoor connected spaces. Two enclosure types were excluded from formal analysis as they were rarely reported: ‘Outside with evening visitor access’ enclosures (n = 3) and ‘Brooder boxes’ used for housing very young or sick animals (n = 2). Kiwi diet (where provided) was characterised using the recipe provided by the facility. Diet components, such as meat and vegetables/fruit, were calculated as a proportion of total fresh food weight.

Qualitative data on behavioural and health problems were provided by facilities as free-text. Responses were sorted into categories. Behavioural problems were categorised as stereotypical/stress-related (e.g. pacing, blowing bubbles when handled), reproductive, quiet/shy, unusual behaviour (behaviour which did not appear to fit with current ideas of stereotypical behaviour in kiwi, but which keepers found to be unexplainable or strange), failure to adjust to nocturnal house (e.g. refusing to eat or leave burrow while in nocturnal house), and human-directed aggression. Health problems were categorised into obesity, reproductive issues, eye issues, foreign body/metal ingestion, respiratory issues, and fungal/parasite infections.

Data were imported into RStudio (Posit team 2024) for analysis. Data were explored and descriptive statistics generated using the R packages ‘psych’ (Revelle 2022), and ‘ggplot2’ (Wickham *et al.* 2016). To test the effect of location (within NZ, elsewhere) on bird age, ANOVA was performed on log-transformed data. Forward stepwise modelling was used to determine the effect of independent variables of interest on bird weight: sex, facility location, enclosure type, enclosure size, number of feeds per day, the presence/absence of reported stereotypical/stress-related behaviour, and the reported presence/absence of health conditions. The same approach was taken to determine the effect of independent variables on enclosure size: facility location, number of kiwi in enclosure, and enclosure type. Chicks, juveniles, and subadults were excluded from this analysis as they are still growing. To account for the dependence among kiwi within the same institution, linear mixed models were attempted with facility included as a random effect using R packages ‘lme4’ (Bates *et* *al.* 2015), ‘lmerTest’ (Kuznetsova *et al.* 2017), and ‘performance’ (Lüdecke *et al.* 2021), however model fit measures, such as AIC and singularity warnings, indicated no improvement over general linear models, which were retained as the most parsimonious model.

Model fit and assumptions were checked using visual inspection of model plots (e.g. QQ plots) and these, along with AIC and likelihood ratio tests, were used to determine which independent variables should be retained, and adjusted means were provided using R package ‘emmeans’ (Lenth *et al.* 2019). Both weight and enclosure size were log-transformed to meet model assumptions. Back-transformed means with 95% confidence intervals are presented.

A Chi-squared test with *P*-value set at 0.05 was used to test the relationship between enclosure type and the presence/absence of behavioural problems and enclosure type, and between the proportion of kiwi housed in nocturnal houses vs off-display within NZ and outside (other enclosure types, n = 3, were removed from analysis).

## Results

### Questionnaire responses

Thirteen of the 14 NZ facilities holding NIB kiwi completed the questionnaire (92.9% response rate), representing 97 of the 107 kiwi (90.7%) held in NZ at the time. Ten of the 17 zoos outside of NZ (58.8% response rate), holding 40 of 48 kiwi (83.3%) completed the questionnaire. Four of the kiwi being held were not NIB, with one being a great spotted (*Apteryx haastii*) and three rowi (*A. rowi*).

### Kiwi demographics

**Figure 1.**
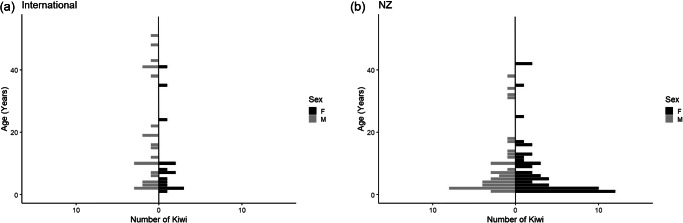
Population pyramid demonstrating the age-sex distribution of the captive kiwi population (a) outside of (n = 40) and (b) within New Zealand (n = 97). Five kiwi (one outside of and four within NZ) of unknown sex at the time of the questionnaire were not included in this figure.

The kiwi reported in the study ranged in age from one month to 50 years old (Figure 1). The mean (± 95% Cl) age of birds in NZ was 7.4 years (5.1, 9.7), which was significantly younger than the mean age of 14.3 years (10.8, 17.9) of those outside NZ (*F*_1,135_ = 8.9; *P* = 0.003). The population outside of NZ had a higher proportion of adults (67.5 vs 53.6%) and a lower proportion of chicks than that within NZ (5 vs 18.5%). Juvenile proportions in both populations were 27–28%.

Overall, there were 68 female kiwi, 64 male, and five of unknown sex, one outside and four within NZ. Of those birds of known sex in facilities outside of NZ, 61.5% (n = 24/39) were male. In contrast, only 43.0% of birds within NZ were male (n = 40/93) (Figure 1).

### Weight

Adult kiwi weight was affected by both sex (*F*_1,74_ = 17.5; *P* < 0.001) and location (*F*_1,74_ = 4.6; *P* = 0.035). Adult females were heavier (back-transformed mean, 95% CIs, 2.5 kg [2.3–2.6]) than males (2.1 kg [2.0–2.2]), and adult kiwi in facilities outside of NZ (2.4 kg [2.2–2.5]) were heavier than those within (2.2 kg [2.0–2.3]). No other statistical effect was found for variables tested.

### Housing

Of the 137 birds included in the questionnaire, three (2.2%) were held outside with evening visitor access (on display to visitors after dusk), 43 (31.4%) were held in nocturnal houses, and 89 (65.0%) were in off-display enclosures (Table 1). Of the kiwi held in off-display enclosures, two (2.3%) were in indoor brooder boxes (~ 1 m^2^), 12 (13.5%) were in a combined indoor and outdoor area, and the remaining 75 (84.3%) were in an enclosure that was outdoor only. Overall, 78 birds (57.0%) were held with another bird (39 pairs), and 59 birds were housed alone (43.0%). Sixty-six NZ birds (68.0% of NZ birds) and 12 birds outside of NZ (30.0% of the total of these) were housed in pairs.

**Table 1.** Number of individual captive kiwi held in different types of enclosures outside of (n = 40) and within New Zealand (n = 97) facilities. Numbers in brackets denote the number of enclosures in which birds were kept

Facility ID	Total	Nocturnal Houses	Off-display	Other housing
I01	3	1 (1)	2 (1)	
I02	11		9 (9)	2 (2) brooder
I03	1		1 (1)	
I04	3		3 (3)	
I05	1	1 (1)		
I06	4		4 (2)	
I07	10		10 (9)	
I08	4	1 (1)	3 (2)	
I09	1		1 (1)	
I10	2	2 (1)		
NZ01	6	4 (2)	2 (1)	
NZ02	1	1 (1)		
NZ03	2	2 (1)		
NZ04	3	2 (1)		1 (1) outdoors on display
NZ05	10	2 (1)	8 (5)	
NZ06	29	6 (3)	23 (13)	
NZ07	4	2 (2)		2 (1) outdoors on display
NZ08	8	4 (4)	4 (2)	
NZ09	7	3 (3)	4 (3)	
NZ10	2	2 (1)		
NZ11	10	4 (2)	6 (4)	
NZ12	2	2 (1)		
NZ13	13	4 (4)	9 (8)	
Total	137	43 (30)	89 (64)	5 (4)

Kiwi included in this study were housed in 98 enclosures. Thirty enclosures were in nocturnal houses (30.3%), two (2.0%) were outside on display, and the remaining 66 enclosures (66.7%) were off-display. As NIB kiwi are not known to leave their burrows during the day (except as a result of sickness or drought), enclosures were categorised as off-display even if they are viewable to visitors during facility opening hours. Of the enclosures categorised as off-display, two (3.0%) were brooder boxes, 12 (18.2%) were combined indoor/outdoor with no visitor access, 38 (57.6%) were outside with no visitor access, 13 (19.7%) were outside where visitors walked past, and one (1.5%) was a visitor walk-through.

At the facility level, 17 of 23 facilities (73.9%) reported holding kiwi in nocturnal houses. In NZ, all 13 facilities reported at least one nocturnal house; one facility had two houses, and one facility had three. Nocturnal houses within NZ contained between one and six separate enclosures, whereas only four of the ten facilities outside of NZ (40.0%) held kiwi in nocturnal enclosures/houses. A higher proportion of kiwi within NZ (38/94, 40.4%) were housed in nocturnal houses (rather than off-display) than in those facilities outside of NZ (5/40; 12.5% (x^2^ = 8.8, df = 1; *P* = 0.003).

**Figure 2.**
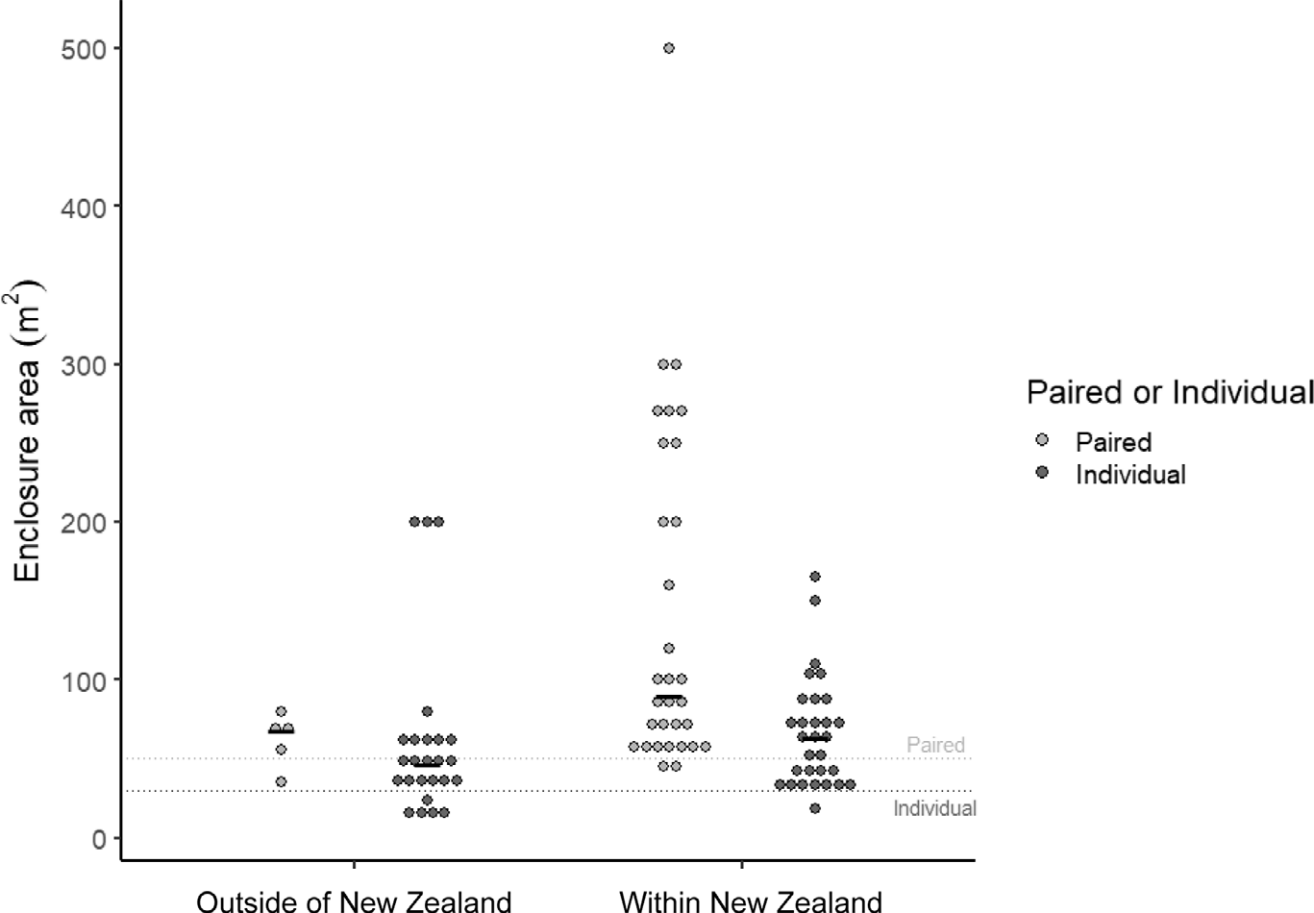
**Enclosure sizes in facilities outside of (n = 32) and within New Zealand (n = 62) housing birds as a pair or individually. Dashed lines indicate recommendations of 30 m** ^**2**^ **for individual kiwi (heavy dash) and 50 m** ^**2**^ **for paired (light dash) outlined in the *Kiwi Husbandry Manual* (Fraser & Johnson 2015). Solid dash indicates mean enclosure size. Two brooder boxes (outside of NZ off-display) of 1 m** ^**2**^ **and two outliers (NZ off-display) of 1,000m** ^**2**^ **removed.**

Enclosure size was influenced by facility location, enclosure type and whether it held a single or paired kiwi. Enclosures in which kiwi were housed in pairs were larger (74.1 m^2^ [95% CI 58.7, 93.6]) than those housing single birds (43.4 m^2^ [36.6, 51.4]; *F*_1,87_ = 16.4; *P* < 0.001; Figure 2). Nocturnal house enclosures (40.1 m^2^ [31, 51.8]) were smaller than off-display enclosures (80.2 m^2^ [69.0, 93.3]; *F*_1,87_ = 23.8; *P* < 0.001). Enclosures outside of NZ (42.3 m^2^ [32.5, 55.0]) were smaller than those within NZ (76.0 m^2^ [65.5, 88.3]; *F*_1,87_ = 15.7; *P* < 0.001; Figure 2).

Nest-boxes were provided for all but five kiwi (Table 2), all of whom were from one facility. Most enclosures had two nesting boxes. Paired kiwi were provided with at least two nest-boxes.

**Table 2.** Number of nest-boxes provided in enclosures (n = 96) for captive kiwi housed in pairs or individually. Percentages of kiwi (by individual or pair) in brackets. Brooder box kiwi (n = 2) not included

Number of nest-boxes in enclosure	Individual kiwi	Pair of kiwi	Total
0	5 (8.8%)	0 (0.0%)	5 (5.2%)
1	24 (42.1%)	0 (0.0%)	24 (25.0%)
2	21 (36.8%)	23 (59.0%)	44 (45.8%)
3	2 (3.5%)	7 (17.9%)	9 (9.4%)
4	4 (7.0%)	6 (15.4%)	10 (10.4%)
5	0 (0.0%)	1 (2.6%)	1 (1.0%)
6	1 (1.8%)	2 (5.1%)	3 (3.1%)

#### Enrichment

**Figure 3.**
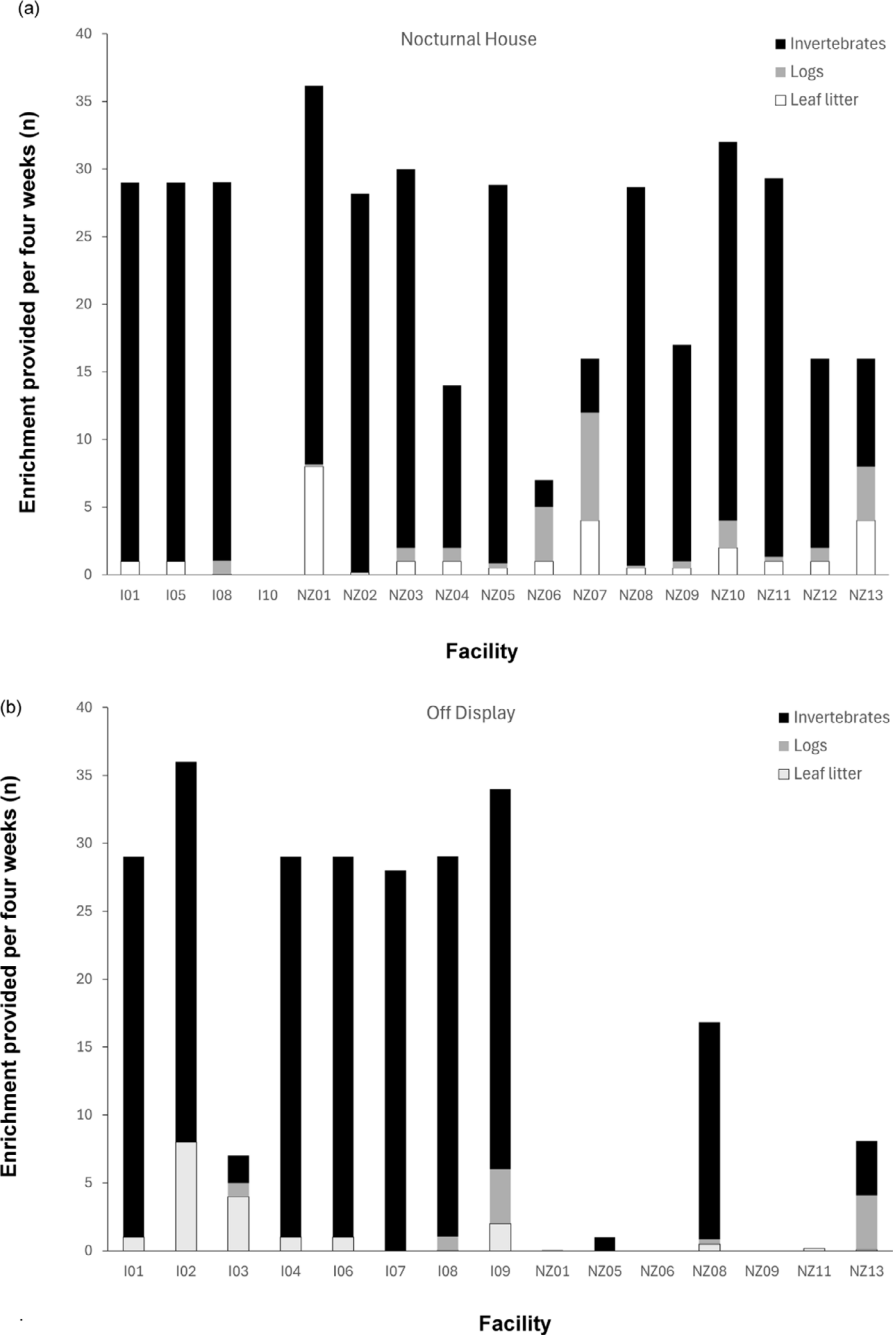
Frequency of different types of enrichment reportedly provided for captive kiwi per four weeks in the enclosures for facilities outside of (n = 38) and within New Zealand (n = 94) located in (a) nocturnal houses (n = 43) and (b) off-display (n = 89). Kiwi housed in brooder boxes (outside of NZ, n = 2) and on-display after dark (NZ, n = 3) not included.

All kiwi living in nocturnal houses, with the exception of one facility, were provided with some form of enrichment (Figure 3). This comprised items such as new leaf litter, logs and invertebrates, including earthworms/night crawlers. The remaining facility provided artificial leaves instead of natural leaf litter.

The frequency of providing each type of enrichment in nocturnal houses varied among facilities. Thirteen facilities (76.4% of facilities with nocturnal houses) added logs as enrichment, and 16 (94.1%) also added some form of invertebrate, 14 of which (87.5%) provided them multiple times a week. Six facilities (35.3%) also reported using other types of enrichment including created devices (made of fronds, plants and grubs), seasonal native berries, toys and scents. Non-natural forms of enrichment (scent lures, toys) were only reported in zoos outside of NZ.

While enrichment was provided to some off-display kiwi (Figure 3), most facilities reported allowing the environment to provide enrichment naturally (e.g. leaf litter falling from planted trees) and then supplementing as necessary. Five facilities never provided leaf litter (33.3% of facilities with outdoor enclosures), and nine never provided logs (60%). Four facilities (26.7%) never added invertebrates to their outdoor enclosures, while nine added them at least once a week (60%). Seven (46.7%) facilities also provided additional enrichment in the form of spices, herbs, pet toys, plastic tubes and forage boxes. As for nocturnal house enrichment, some zoos outside of NZ provided this kind of ‘unnatural’ enrichment, but none within NZ did.

### Nocturnal houses

#### Age of birds

The mean (± SD) age of kiwi in nocturnal houses was 5.1 (± 8.7) years. Of the 43 birds in nocturnal houses, 17 (32.6%) were over the age of three. Thirteen of these were within NZ, and three outside.

#### Temperature and humidity

The air temperature inside nocturnal houses was regulated in all four facilities outside of NZ, but only in ten houses (62.5% of houses in NZ) in seven NZ facilities. In regulated houses, there was no consistency in the set temperature, which ranged from 12 to 24°C with a mean (± SD) of 17.9 (± 3.6)°C. Only six facilities, comprising nine nocturnal houses, (four in NZ, two outside of NZ) controlled relative humidity levels, with set levels ranging from 50 to 85% with a mean of 67.6 (± 12.6)%.

#### Light

The lighting regime was reversed in all nocturnal houses. In eight nocturnal houses (40%; 7/16 within NZ, 1/4 outside), a dawn/dusk timer was used with a set period of either intermediate light intensity or gradual change. In the other 12 nocturnal houses (60%; 9/16 within NZ, 3/4 outside), lighting went straight from dark to light and *vice versa.* Lighting in 15 of the 20 nocturnal houses (75%; 12/16 within NZ, 3/4 outside) was seasonally adjusted for the duration of night. Five (25.0%) of these were specifically changed to reflect the natural hours of night. Thirteen facilities (14 houses; 70%) only adjusted the time due to daylight savings. In facilities where the length of night was not changed seasonally, night length varied from seven to 13 h, with a mean of 9 h 44 min (± 1 h 50 min). Visible lighting colour (according to the human eye) during simulated night-time was highly variable with no consistency among facilities.

#### Floor substrate

Most facilities (15/17 facilities; 88.2%) used leaf litter as the floor substrate for nocturnal houses; the remaining two used dried fern fronds (one within NZ; 5.9%) or artificial leaves (one outside NZ; 5.9%). Leaf litter was sourced from native forest outside the facility (seven in NZ; 46.7%), within the facility (two outside of NZ, five within; 46.7%) or bought already sterilised (one outside of NZ; 6.6%). Litter sourced naturally was stored before use for less than 48 h in all facilities. One facility outside of NZ (6.6%) reported they froze their leaf litter for three days prior to use. Three facilities (20.0%) added it at least once a week and seven (46.7%) at least once a month. Four (26.7%) added litter less than once per three months and one (6.6%) only every two years. Prior to use, all naturally sourced leaf litter was checked for metal and other debris via visual inspection (four facilities; 28.6%), a metal detector (nine; 64.3%), a magnet (four; 28.6%), and/or manual sieving (one; 7.1%).

The entire floor substrate in nocturnal houses (subsoil as well as any leaf litter) was completely replaced on a regular basis in seven facilities with nocturnal houses (42.7%): this occurred every six months in one facility (5.9%), every year in another (5.9%), every two years in four facilities (23.6%), and every three years in one (5.9%). Three facilities (17.6%) replaced substrate only when new birds arrived, and seven others (41.2%) reported never having replaced the whole substrate and that there were no plans to do so.

#### Barriers

All 20 nocturnal houses were designed so as to enable visitors to view kiwi, but the form of barrier between the kiwi and visitors varied. Two houses (10.0%) had only a partial barrier of wood or glass that reached from the floor to adult human waist-to-head height. One (5.0%) reached above adult head height but not to the ceiling. Thirteen houses had a glass barrier that reached up to the ceiling with either single- (n = 6; 30.0%), double- (n = 3; 15.0%) or triple-glazed glass (n = 3; 15.0%). Another (5.0%) had single-glazed glass to the ceiling for most of the enclosure but included an open viewing area as well as a barrier reaching to adult waist height.

#### Sound

Six of the facilities with nocturnal houses (35.3%) provided an artificial soundscape in their nocturnal house, but one of these applied sound in only one of their three nocturnal houses. In the five facilities within NZ with soundscapes, the sound provided included wind, kiwi calls, ruru/morepork (*Ninox novaeseelandiae*) calls, running water, crickets, and frogs. The one facility outside of NZ using a soundscape played African bird sounds.

### Off-display enclosures

Fifteen facilities (65%) reported keeping some kiwi off-display (Table 1). All 36 in NZ and 16 of the 28 off-display enclosures outside of the country were entirely outdoors (81.3%). Twelve of the off-display enclosures outside of NZ (18.8%) allowed kiwi access to both indoors and outdoors. Two further off-display enclosures were brooder boxes.

All outdoor, off-display enclosures were planted with grass and trees in soil, while indoor off-display enclosures were filled with soil and leaf litter. The size of off-display enclosures ranged from 11 m^2^ (for a juvenile pen) to 500 m^2^, with a mean (± SD) of 106.1 (± 93.0) m^2^. The largest enclosure reported was a multi-species outdoor walk-through enclosure within NZ.

#### Location and barriers

Most off-display enclosures (not designed for kiwi viewing by visitors, as discussed in *Housing*) were reported to be in areas of the facility to which visitors had no physical access (51 of 64 enclosures; 79.7%). Eleven enclosures (17.2%) that visitors could walk past had barriers (fencing) either to adult waist height (8; 12.5%) or above adult head height (3; 4.7%), with an additional one not providing barrier type. The remaining off-display enclosure was the visitor walk-through with a waist-high rail that kiwi could move beneath.

### Kiwi management

#### Diet and feeding

All the facilities within NZ fed their kiwi a variation of the ‘Massey diet’, which was developed at Massey University, NZ and has a base (53.5%) of lean beef and ox heart (Fraser & Johnson 2015). Seven of these NZ facilities (53.8%) reported feeding the ‘Massey’ diet as prescribed, while the other six reported using substitutions such as different oils or adding calcium powder. Zoos outside of NZ reported using a wide variety of recipes and supplements. Diet composition ranged from 27.0 to 83.0% meat (estimated mean 52 [± 16.5]%), and 17.0 to 58.0% fruit/vegetables (31.4 [± 12.7]%). Commercially prepared cat or dog food and avian food mixes were also used by a number of the facilities outside of NZ.

Most facilities used more than one method of feeding the main diet. In NZ, bowls were used in eleven (84.6%) facilities, test tubes pushed into the ground in five (38.5%) and cups in two (15.4%). In facilities elsewhere, test tubes were used in two establishments (20%), and bowls in another nine (90%). Plus, in three other facilities outside of NZ (30%) keepers also fed kiwi by hand.

#### Health checks and weighing

In all the facilities in NZ, all birds in nocturnal houses were visually checked every day. Off-display birds were checked visually every day in five of seven facilities (71.4%) and visual checks were undertaken twice weekly in the remaining two facilities. Outside of NZ, nine of ten facilities checked their birds visually at least once daily (90%), while one checked them weekly.

Regular physical checks (where birds were caught and handled) by keepers were carried out at 12 of the 13 facilities in NZ (92.3%): weekly at one facility, monthly at five, quarterly at two, and six-monthly at four. The remaining facility did not specify how often physical health checks were conducted. Additionally, at one of these NZ facilities, chicks were physically checked and weighed daily, and any ‘young birds’ moved outside were weighed and physically checked fortnightly. Weight was measured weekly at one facility, monthly at five, every two months at two, quarterly at one, and six-monthly at three. Some form of body condition score was used at eleven of the 13 facilities (84.6%). Four reported measuring food intake as part of the method for monitoring physical condition.

In the ten facilities outside of NZ, physical checks by keepers, including gait observation, were conducted weekly at one and quarterly at another. The eight remaining facilities did not report physical checks outside of weighing the birds, which was undertaken weekly at five, monthly at three or quarterly at one. Six of these ten facilities reported monitoring using some form of body condition score.

Kiwi were veterinary checked six-monthly at four of 13 NZ facilities (30.8%) and annually at one (7.7%). In eight facilities (61.5%), veterinary checks were only performed due to health concerns, when being transferred to another facility, or in preparation for release, as recommended in the *Manual* (Fraser & Johnson 2015). Elsewhere in the world, veterinary checks were undertaken every two months in one facility and included radiographs. Five other facilities (50.0%) reported annual veterinary checks, with one of these additionally performing a full diagnostic examination (radiographs, anaesthesia) every three years. Four of these facilities outside of NZ (40.0%) carried out veterinary checks only when sickness was suspected.

#### Health issues

There were 60 cases of health issues reported in the survey. These included mouth infections/bill injuries (13), eggbound/reproductive issues (eight fixed), eye concerns (five fixed, one ongoing), metal ingestion (five fixed), respiratory issues (three fixed), and parasitic or fungal infections (two fixed). Other kiwi (one incident per condition) were reported as having a heart condition, elevated lead levels, yolk removal as a chick, a calcium deficiency after laying, salmonella, feather loss, salivary gland adenoma or torticollis, all of which were reported as resolved. Ongoing health issues included deformities to eyes, bills or legs (six), blood disorder (one), a heart murmur (one), and obesity (one resolved, ten ongoing). In the NZ facilities, health issues were reported in 56 of 97 kiwi (57.7%) and, outside of NZ, in 13 of 40 birds (32.5%). Five kiwi were reported to have had more than one type of health issue. Health issues were reported in 25 of the 68 female birds (36.8%) and 27 of the 64 male birds (42.2%).

**Figure 4.**
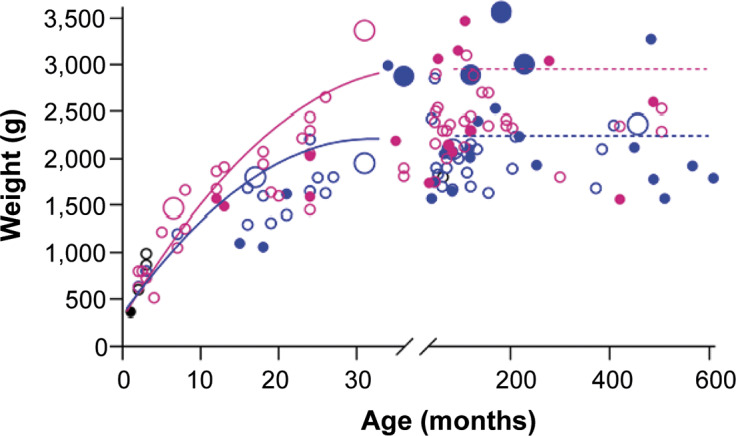
Age and reported weight of male and female captive kiwi within New Zealand (NZ; n = 97) and elsewhere in the world (n = 40). The lines represent the mean weight of adult kiwi reported in the *Kiwi Husbandry Manual.* Pink circle = female, blue circle = male, black = unknown sex. Closed circle = Outside NZ, Open circle = Within NZ. Large circles indicate those birds reported as overweight/obese by their facility.

Obesity was reported to be current in ten kiwi, with six in NZ (6.2%) and four in facilities elsewhere (10%) (Figure 4). Five of these were adult males, three were subadult males, one was a subadult female, and one was a female juvenile. Other individuals were not reported as overweight but had reported weights well above the mean for their sex.

#### Behavioural issues

Within NZ facilities, 23 of 97 birds (23.7%) were reported to have a behavioural issue or had shown one in the past (Table 3). Keepers in facilities elsewhere reported behavioural issues in five of 40 birds (12.5%), all of which were current. Two kiwi, displaying human-directed aggression or stereotypical pacing, were reported as increasing these behaviours during the breeding season. Overall, eleven of the 68 female birds (16.2%) and 17 of the 64 male birds (26.6%) were reported to have had a behavioural issue at some point of their captive life. Stereotypical behaviour was reported in one juvenile (out of 20), six subadults (out of 38) and five adults (out of 79). Kiwi currently living in a nocturnal house (14/43; 24.6%) were more likely to be reported as having a behavioural problem than those off-display (13/89; 14.3% (x^2^ = 5.0, df = 1; *P* = 0.026).

**Table 3.** Number of captive kiwi reported to have one or more behavioural problems in NZ (n = 23 of 97) and outside of NZ (n = 5 of 40), and examples of reported behaviours. Each number represents one reported issue, with some individuals being reported to have more than one problem

Behaviour	Example as reported	Number
Stereotypical/stress-related behaviour	Pacing, blowing bubbles while handled	13
Reproductive	Egg smashing, chick killing, disinterest in mating, incubating stones, pining for mate	6
Quiet/Shy		4
Unusual behaviour	Clumsy, falling on side, with no medical explanation	2
Failing to adjust to nocturnal house	Lack of feeding or failing to come out of burrow in nocturnal house	3^a^
Human directed aggression		1
Total		29

a Two of these kiwi had been removed from the nocturnal house to off-display enclosures at the time of the questionnaire and were no longer displaying behavioural issues

## Discussion

This study aimed to describe the captive kiwi population within and outside New Zealand, including their demographics, reported health issues and behavioural concerns, to describe current housing and husbandry practices for kiwi, and to explore associations between enclosure type and reported behaviour problems. The information gathered in this survey represents almost all facilities holding kiwi in New Zealand and most birds held in captivity at the time of the survey. While only around 60% of those facilities outside of NZ holding kiwi responded, these accounted for more than 80% of birds held overseas. Thus, the findings discussed below accurately represent the current management and environment of captive kiwi in New Zealand and around the world.

### Kiwi population demographics, health and welfare

On average, the population of kiwi held outside of NZ was almost twice as old as that within the country. This was because of a higher proportion of adults and a lower proportion of juveniles in facilities beyond NZ. This is likely due to the NZ procedure of releasing birds at breeding age or when the bird’s genetics are well represented in the captive population (Barlow 2018). As their genetic presence in the population (mean kinship) determines release, birds can be released at any age. NZ also has access to new birds through the national egg-hatching/chick-rearing programme (Operation Nest Egg), enabling the population to add new founder birds and maintain a high level of genetic diversity. At the same time, it is unlikely that any further birds will be exported from NZ to facilities elsewhere due to consideration of the cultural expectations of NZ’s first peoples and because of the views of NZ public following recent treatment of kiwi held in international zoos (McClure 2023; Wilton 2024). This could have long-term ramifications for the population outside of NZ, as the population is too small to maintain 90% genetic diversity over 200 years; the gold standard for captive population genetic management (Soulé *et al.* 1986). A loss of genetic diversity can lead to inbreeding depression, resulting in loss of fertility and reduced offspring survival (Charlesworth & Willis 2009).

### Weight and body condition

Consistent with Robertson *et al.* (2017), female kiwi were heavier than males. The average weights were somewhat lower than those reported in the *Manual* (males 2.1 vs 2.25 kg, females 2.5 vs 2.75 kg). Kiwi in facilities outside of NZ were heavier than those within, possibly due to differing population geographic origins; birds outside of NZ are primarily from the Northland taxa. In contrast, NZ facilities traditionally hold Eastern NIB, which are suggested to be lighter (Burns 2014). However, other published data do not support this, with NIB weights overlapping between taxa (McLennan *et al.* 2004; Scofield & Stephenson 2013). These studies were conducted with limited sample sizes in one geographic area and, as such, may not be accurate to the taxa as a whole. These data mean that we need to fully understand the weight differences between NIB taxa and how it is affected by captivity.

Around 7% of birds in this study were reported as being overweight or obese by keepers; eight were males, and the distribution was roughly equal across locations. However, other birds weighed more than those reported to be obese. This included two males in facilities outside of NZ over 2.9 kg, which is markedly heavier than the published range. It is unclear whether the birds are larger and have good body condition or if the keepers misjudge what constitutes an overweight kiwi. Nearly three-quarters of facilities used some form of body condition scoring for kiwi based on feeling the ribs, hips or spine (Morgan 2008). However, the lack of standardised guidelines for body condition scoring of kiwi could hinder accurate weight management leading to poorer health outcomes.

Interestingly, while enclosures in facilities outside of NZ were significantly smaller than those in NZ, enclosure size did not significantly influence kiwi weight. Other factors are likely also at play. For example, diet could also play a role, with diets outside NZ differing from facility to facility as well as from the more standardised NZ diet (to be discussed later).

### Illness and injury

Health issues were reported in 58% of NZ kiwi and 33% of those elsewhere. Of the reported health issues, over 20% were mouth infections or bill injuries, 12% were reproductive issues, while other issues included metal ingestion, eye concerns, respiratory issues, and parasitic/fungal infections. Health problems were more commonly reported in NZ facilities, with 60% of birds experiencing issues, possibly due to some coming from rehabilitation facilities or vets with conditions that prevent their release into the wild. However, the standard procedure is that these birds are euthanased (Barlow 2018). It is unclear how many of these birds remain in captivity. Since the data were collected retrospectively for each bird, the housing and environmental conditions at the time of each health issue are unknown, preventing any conclusions being drawn regarding potential associations. As far as the authors are aware, there are no studies into the overall sickness/injury rates of captive or wild kiwi.

### Health checks

Visual health checks were conducted daily in all nocturnal houses and daily or twice weekly in most off-display enclosures. Physical checks, including weighing and body condition scoring, occurred monthly to every six months in NZ facilities, with more frequent checks for chicks. Facilities outside of NZ primarily conducted weighing but did not report regular physical health checks. One-third of NZ facilities had annual vet checks, as did half of those elsewhere. The remaining facilities only conducted vet checks as required by the *Manual* – when birds were being exported to another facility or showing signs of sickness.

According to the 2^nd^ minimum standard of the NZ Code of Welfare: Zoos, some form of daily inspection of animals must be conducted (New Zealand Ministry for Primary Industries 2018). This is especially important in avian species, which may hide illness due to predator avoidance in the wild (Morgan 2008; Schulte & Rupley 2004; Weber 2011), which could delay diagnosis if health checks are less frequent. However, more frequent handling may increase stress and impair immunity or could lead to weight loss (Lilliendahl 1997). For example, low levels of parasitic or fungal spores in kiwi enclosures, such as *Aspergillus* spp, can be present without causing illness (Glare *et al.* 2014), but stress can make kiwi more susceptible. The survey did not allow for exploration of the relationship between health check frequency and illness or death rates, thus no conclusions can be drawn on the impact of health check frequency on kiwi health.

#### Behavioural concerns

Nearly one-quarter of kiwi in NZ were reported to have or have had a behavioural issue, almost double the percentage seen in facilities elsewhere in the world, despite the latter’s older population and smaller enclosures. The most common behavioural issue was stereotypical/stress-related behaviour, primarily in subadult and adult birds, but also seen in younger chicks. Stereotypical pacing was also reported by Wesley & Brader (2014) for a juvenile kiwi kept in an inside pen on a natural lighting schedule.

We found that over one-third of kiwi held in nocturnal houses were adults and most of these were in NZ facilities. This is not consistent with the Kiwi Captive Management plan (Barlow 2018) which recommends that only young kiwi under the age of three are housed in nocturnal houses in the hope of reducing pacing behaviour and egg-binding. However, the presence of stereotypical behaviour in chicks and juveniles suggests that removing adult birds from nocturnal houses does not fully address behavioural problems. The ongoing presence of stereotypical behaviour in kiwi indicates that the environment/husbandry procedures are not yet optimal and warrant further research.

The higher number of adults in nocturnal houses in NZ may partly explain why behavioural problems were more commonly reported in these birds compared to those in outdoor enclosures. However, behavioural issues in outdoor enclosures may be underreported since kiwi are naturally inactive during opening and staffed hours and, as such, less frequently observed. Even in nocturnal houses, reports depend upon keepers’ ability to observe birds amidst busy schedules (Margulis & Westhus 2008). Further research should be conducted into the 24-h behaviour of kiwi in both nocturnal houses and off-display to better understand how a reversed light cycle and nocturnal house environment affect captive kiwi behaviour and welfare (Brando & Buchanan-Smith 2018).

Other factors impacting behavioural issues could involve enclosure size, more frequent human-animal interactions, increased noise levels, reversed light cycle, or environmental features. These variables were unable to be tested against behavioural issues in this study due to small sample sizes and high levels of variation.

### Housing and management practices consistent with the *Kiwi Husbandry Manual*

The *Manual* provides recommendations and minimum standards based on current best practices. The data here show that many facilities follow the *Manual*’s standards for aspects of kiwi housing and management, particularly enclosure size, enrichment, and lighting in nocturnal houses. However, consistency was lacking in barriers, humidity, substrate type/replacement, diet, and feeding.

#### Housing

Approximately one-third of kiwi were kept in nocturnal houses with a reversed light cycle for visitor display. All New Zealand facilities house kiwi in nocturnal houses (average of 2.4 kiwi per house), while only seven use off-display enclosures for breeding or release preparation. Four of ten facilities outside of NZ display kiwi in nocturnal houses, but 90% of kiwi held outside of NZ are housed off-display. Off-display kiwi could support the international breeding programme, but those in international facilities are unlikely to contribute to NZ’s wild populations.

#### Enclosure size and pair housing

Most NZ enclosures exceeded the minimum size limits noted in the *Manual* (30 m² for one bird, 50 m² for two), except for one nursery enclosure (one kiwi) and three paired enclosures (six kiwi; two nocturnal houses, one off-display). Of the 32 enclosures outside of NZ, two nocturnal house enclosures were below the recommended sizes (both individually housed kiwi), as were four off-display enclosures (five kiwi; one pair, three housed singly). Off-display enclosures were roughly twice as large, on average, as nocturnal house enclosures, and enclosures holding pairs were larger than those for individually housed birds. Despite having more nocturnal houses, NZ enclosures were nearly twice the size of those in establishments elsewhere.

The origin of the minimum size limits is unclear. Prior to human arrival, kiwi densities were 40–100 adults per 1,000,000 m² (McLennan *et al.* 1996), whereas a kiwi pair’s territory now ranges from 20,000 to 1,000,000 m² (Fraser & Johnson 2015; Holzapfel et al. 2008), much greater than the space provided by the *Manual.* Wild kiwi use the space for foraging and changing den sites nightly (though sometimes returning to sites used previously) (McLennan *et al.* 1987; Taborsky & Taborsky 1995). Further, juvenile kiwi use different types of habitat depending on the season (e.g. pasture in autumn, seral vegetation in winter), indicating the value of being able to access to varied habitat types (Gibbs 2000; Gibbs & Clout 2003). Both the inability to change den sites and the lack of variability in habitat type in captivity could have an impact on kiwi welfare. Additionally, as nocturnal house enclosures are significantly smaller than outdoor enclosures, this size limit could play a factor in the increase in behavioural problems seen in nocturnal houses. As NZ has more nocturnal houses than elsewhere, this could also play a role in NZ having more behavioural problems reported than in other parts of the world.

This study found that nearly 60% of kiwi are housed in pairs, more commonly within NZ compared to outside. Kiwi are territorial, with birds of some taxa killing other individuals that encroach into their territory (Colbourne 2002; Colbourne & Kleinpaste 1983; Corfield *et* *al.* 2008; Taborsky & Taborsky 1992). While individuals or pairs will maintain a territory, kiwi pairs do not spend all their time together. For example, pairs in Hawkes Bay were found to share dens on only 8% of days, increasing to 14% during the breeding season (Miles *et al.* 1997). In captivity, kiwi are forced to spend all their time relatively near each other and often near other pairs. Kiwi housed in pairs would have limited options for retreat, however, all pairs of kiwi had access to at least two nesting boxes, so each bird would have the opportunity to den separately. These forced interactions may increase stress experienced by captive kiwi, which may contribute to the finding that behavioural issues are more often reported in NZ facilities, where more kiwi are held in pairs (68%) than in other parts of the world (30%). The presence of conspecific neighbours for mainly solitary species (Okapi [*Okapia johnstoni*] and black rhino [*Diceros bicornis*]) in captivity has been shown to increase stereotypical behaviour and decrease breeding success (Bennett et al. 2015; Carlstead *et* *al.* 1999; Whitham & Miller 2019). Furthermore, singly housed individuals can call and respond to a kiwi of the opposite sex but are unable to get to them. This could lead to frustration and diminished welfare.

#### Enrichment

We found that enrichment was commonly provided in nocturnal houses, primarily in the form of leaf litter and invertebrates. All facilities (bar three) provide invertebrates to their nocturnal house enclosures at least twice a week. Invertebrates were added once a week and once a fortnight in two further enclosures, with only one facility not reporting the addition of enrichment to their nocturnal house. Enrichment was provided less often in off-display enclosures, likely because the natural environment was believed to provide the same enrichment opportunities. Non-natural forms of enrichment, such as PVC piping to run through, dog and cat toys, and scent enrichment, were only reported in facilities outside of NZ. This may reflect the fact that these facilities do not have to be consider kiwi needing to retain natural behaviour for wild release and so they can interact more with humans and be offered non-natural enrichment. The use of non-natural enrichment could allow for a far greater range of enrichment ideas and should be researched further to determine if such approaches could improve kiwi welfare while maintaining natural behaviour for release.

#### Lighting

Kiwi in nocturnal houses are kept under reverse lighting conditions, with lights simulating daytime during the natural dark phase and nocturnal lighting during the day (Fraser & Johnson 2015). In this study, light transitions were reported to be abrupt in most nocturnal houses, with only eight of 20 implementing gradual changes to simulate dusk and dawn. The length of the ‘night’ period varied from 7 to 13 h, and only three-quarters of facilities adjusted night length for seasonal changes, with the remainder using static light hours except for daylight saving.

The *Manual* suggests 8–10 h of darkness to increase kiwi activity for visitors. However, the night length in Hawkes Bay, where Eastern NIB are found, ranges from 12 h 38 min at the winter equinox to 6 h 37 min at the summer equinox (Astronomical Applications Dept 2024). While 8–10 h may enhance the visitor experience, it does not replicate the natural environment, potentially contributing to behavioural issues in nocturnal houses. Off-display enclosures with natural light cycles may mitigate this. Additionally, consistent day/night lengths (found in a quarter of facilities) could be monotonous for kiwi, resulting in reduced welfare (Burn 2017). Further, once kiwi are placed outside in off-display enclosures, the natural changes in day length could result in confusion.

Lighting spectra varied across facilities, likely due to the *Manual*’s lack of guidance on ideal lux or spectra for kiwi. Natural light levels vary by location, moon phase, and time of night (Kyba *et al.* 2017; McNaughton *et al.* 2022). In contrast, nocturnal house lighting levels remain constant, which may contribute to behavioural problems occurring more frequently in nocturnal houses due to monotony.

No information was collected on lux levels, flicker rate or lighting type as facilities often do not have access to the equipment to accurately measure these factors. Further research into the effect of these variables on kiwi behaviour and welfare would be beneficial.

#### Temperature

The temperature was regulated in all nocturnal houses in those facilities outside of NZ but only in approximately half of those in NZ, a total of about sixty per cent of nocturnal houses. The *Manual* recommends that temperatures in nocturnal houses remain below 25˚C and ideally range between 14 and 20˚C. In the seven NZ facilities that regulated their nocturnal houses, temperature ranged from 12 to 24˚C. These all fall within the recommendations and the kiwi thermoneutral zone of 10 to 30˚C (McNab 1996). However, some facilities that do not regulate their nocturnal house temperature (and in off-display enclosures) have winter temperatures that can reach below zero, and while estimates based on metabolism and body temperature suggest that the lower thermoneutral range could extend as low as 5˚C (Maloney 2008; McNab 1996), these lower temperatures could still be causing a degree of discomfort for kiwi and compromising both health and welfare.

### Housing and management practices not consistent with the *Kiwi Husbandry Manual*

#### Humidity

In contrast to temperature, only six facilities controlled humidity in their nocturnal houses, with levels ranging from 50 to 95%, averaging 68%. The *Manual* recommends humidity between 50 and 60% to minimise fungal infection risk, suggesting that one-fifth of nocturnal houses exceed this limit. Some facilities cited higher humidity to support plant growth.

High humidity can promote *Aspergillus* spp growth, but while research shows that spores are often present at low levels without causing infection (Glare *et al.* 2014) immunosuppression or other predisposing factors are necessary for disease (Hauck *et al.* 2020). While high humidity may contribute to health issues, other factors are likely involved. Consequently, the impact of humidity on kiwi health remains poorly understood.

#### Barrier type

Sixteen nocturnal houses had full barriers separating kiwi from visitors, while four of the nocturnal houses in NZ used partial barriers (fences/glass) that did not reach the ceiling. The *Manual* recommends solid barriers to reduce noise transmission, and previous research shows that increased noise can increase abnormal behaviours in kiwi (Davison *et al.* 2021) and other avian species (Rose *et al.* 2022). A complete floor-to-ceiling barrier will also have the additional benefit of stopping visitors from interfering with the enclosure. Visitors could throw items into the enclosure that can harm kiwi, such as small pieces of metal (Gulliver *et* *al.* 2022). Visitors reaching into enclosures could also force human-animal interactions that may be aversive to kiwi affective state (Morgan & Tromborg 2007).

As well as visitor sound, the presence of a sound-scape recording could also impact on kiwi behaviour if the sound is perceived by birds inside the enclosure. NZ facilities played a soundscape recording consistent with sounds in native forests, including bird calls, water, and insect/amphibian calls. Such bird calls could have an impact on kiwi behaviour due to their territorial nature. To illustrate, a little spotted kiwi (*Apteryx owenii*) was filmed destroying the nest of a North Island robin (*Petroica longipes*) pair in its territory (Shaw & Mackinlay 2016). In addition, Brainard & Doupe (2002) indicated that certain bird species are able to recognise calls from conspecifics without ever having heard them before. Furthermore, songbirds have been found to repeat the calls they hear as young. While they prefer to learn the songs from conspecifics of the same species, they will learn from other species if conspecifics are not present. As such, the presence of a soundscape could have a negative impact on the ability of young kiwi to produce calls required for mate finding and territory defence and should be researched further.

Fifteen facilities had off-display enclosures, with 51 enclosures inaccessible to visitors. One enclosure was a visitor walk-through aviary with multiple species and a waist-high fence stopping visitors from stepping off the track but allowing bird movement underneath, and the barrier type in one enclosure was not provided. Of the eleven enclosures where visitors could walk past, eight had waist-high barriers, and three had barriers above head height. Kiwi are nocturnal and rarely seen during the day, except in cases like the Stewart Island/Rakiura tokoeka (Southern brown kiwi; *Apteryx australis*) where summer nights are short (Save the kiwi 2024) or the NIB during droughts (Jackson 2019). As such, while housed in visitor-accessible areas, kiwi are still considered ‘off-display’ as they remain in dens during daytime hours. These kiwi may be exposed to more daytime noise than those in off-limits areas. No significant effects of visitor noise on kiwi behaviour or welfare were found, though further research is recommended to assess its impact on sleep and subsequent nocturnal behaviour.

#### Substrate

Two of the 17 facilities did not use leaf litter in their nocturnal houses, opting instead for artificial leaves or dried fern fronds. One facility freezes its leaf litter for three days prior to use, which reduces the risk of infection, though this does not eliminate aspergillus or cryptococcosis (Reed *et al.* 2020). These methods, however, prevent the natural addition of invertebrates within the leaf litter for kiwi enrichment, which could reduce successful foraging attempts for kiwi and increase frustration, leading to poor welfare outcomes.

The remaining facilities followed the *Manual* guidelines, properly storing and screening leaf litter (via visual inspection, sieving, or magnet use) before use in nocturnal enclosures, as Glare *et al.* (2014) recommended. Anecdotal evidence suggests that these measures have reduced the mortality of kiwi from fungal infections, but further research is needed to confirm this.

Only seven of the 17 facilities regularly replaced the substrate in their nocturnal houses, with replacement intervals ranging from every six months to every three years. Three facilities replaced it when new birds arrived, while seven had never replaced the substrate and had no plans to do so. The *Manual* recommends annual substrate replacement to prevent the build-up of contaminants and faecal material. However, such recommendations have yet to be made for off-display enclosures. Research on the prevalence of infectants in un-replaced substrates over time would help determine if this practice is necessary.

#### Diet and feeding

All NZ kiwi were fed the ‘Massey diet,’ but nearly half of the facilities modified the recipe by substituting oils or adding minerals like calcium powder. The Massey diet consists of 53.5% meat. In those participating facilities outside of NZ, diet composition varied greatly, with meat content ranging from 27 to 83%, and no two zoos using the same recipe. While the Massey diet is considered adequate, it is not optimal, and research is ongoing (Jenkinson 2023).

Most places used cups or bowls to feed kiwi however test tubes placed in the ground were used in 35% of the NZ facilities and 20% of those elsewhere. These facilities outside of NZ also report feeding birds by hand which, in NZ, was only ever done for instances of newly hatched chicks not feeding independently (Bassett 2012; Prier *et al.* 2013). The *Manual* recommends using test tubes to simulate probing for invertebrates (Fraser & Johnson 2015). Feeding by hand in zoos outside of NZ may result from a different kiwi-handler relationship than that in New Zealand and the lack of a need for kiwi to retain natural behaviour for release. However, hand feeding could also impact the amount of time kiwi spend foraging, as they will not be seeking to resolve the negative affective state of experiencing hunger. This could also reduce their foraging time, leading to less exercise and potentially contributing to the heavier weights observed in the kiwi population outside of NZ.

Feed times and frequency vary across facilities. Five feed once in the morning before the nocturnal light phase begins, likely to avoid disturbing kiwi when they are out of their dens. Four other facilities feed once during the nocturnal phase. Seven facilities provide food 2–3 times daily. The *Manual* recommends feeding kiwi at least twice daily to support longer foraging periods and provide opportunities for keeper-visitor interactions while kiwi forage. More frequent feedings may lead to kiwi either becoming habituated to human presence, reducing stress, or becoming more sensitive to humans, increasing stress. It could also result in a dependency upon humans for food, reducing natural foraging time and potentially impacting post-release success. The authors are unaware of any research that has explored the impact of kiwi-human or keeper-animal relationships on kiwi welfare or on the long-term post-release success of kiwi.

No data were collected regarding the amount of diet presented to kiwi across facilities. The way pair-housed kiwi are reportedly fed does not allow determination of individual birds’ intake. Further research into the links between feed amount and body condition score would be beneficial.

## Animal welfare implications and conclusion

This study has explored various aspects of kiwi health and welfare in captive settings, both in New Zealand and elsewhere in the world, including demographics, health and behavioural issues, environmental variables and management practices.

Managing kiwi in captivity presents a challenge for facilities, particularly in NZ, as they must ensure the birds’ welfare is maintained while also preparing them for successful survival post-release. Current guidelines are outlined in the *Kiwi Husbandry Manual*, but for many areas of husbandry guidelines are vague or unspecified. Further research is vital to better understand kiwi welfare and to improve the captive environment.

Our study identified key areas for improvement of kiwi health, such as body condition scoring and weight management. Additionally, more research is needed to examine health outcomes in relation to substrate treatment and the frequency of health checks.

Kiwi housed in nocturnal exhibits were more frequently reported as exhibiting behavioural problems. However, it remains unclear whether this is due to reporting bias or specific aspects of the enclosure environment. While no significant associations were found between environmental factors and kiwi behaviour in this study, the findings suggest that variables such as enclosure size, the number of kiwi housed together, lighting levels and night length, and enrichment or foraging opportunities may influence these behavioural issues. Further research is essential to better understand these factors, as well as to explore the impact of the captive environment on off-display kiwi.

## Supporting information

Connor et al. supplementary material 1 Connor et al. supplementary material

Connor et al. supplementary material 2 Connor et al. supplementary material

## Supplementary material

The supplementary material for this article can be found at http://doi.org/10.1017/S0962728625100109.
