# Supplementary material for: Survey of husbandry practices and captive environments for North Island brown kiwi (Apteryx mantelli) housed in facilities within and outside New Zealand
Source: Anim Welf. 2025 Jun 20;34:e41. doi: 10.1017/S0962728625100109 (PMC12284840; doi:10.1017/S0962728625100109)
Supplement: Connor et al. supplementary material 1 — Connor et al. supplementary material [file S0962728625100109sup001.pdf]

Survey of husbandry practices and captive environments for North Island brown kiwi *(Apteryx mantelli)* housed in facilities within and outside New Zealand

Rebecca L Connorhttps://orcid.org/0000-0002-5889-3839[1], Nicholas Ling[1], Ngaio Beausoleil[2], Kris Descovich[3], Todd S Jenkinson[4]

[1] Te Aka Mātuatua School of Science, University of Waikato, New Zealand

[2] Animal Welfare Science and Bioethics Centre, School of Veterinary Science, Massey University, New Zealand

[3] School of Veterinary Science, The University of Queensland, Australia

[4] Zoo and Aquarium Association, New Zealand

Author for correspondence: Ngaio Beausoleil, email: N.J.Beausoleil@massey.ac.nz

Appendix A

Questionnaire structure

Facility-level questions focused on the opening hours of the facility, access of visitors to kiwi (open question (o)), diet (o) and the types and frequency of health checks (o). Facilities were asked to provide the frequency of visual, physical and vet health checks. A visual health check is when keepers observe the animal without touching it, while a physical check includes the physical handling and checking of kiwi. A vet check is when a vet is involved in the procedure, as opposed to a keeper or other staff member. Facilities were also asked how often the weight of kiwi was measured. Some facilities also reported the frequency of faecal checks (for parasites), though this was not asked.

Enclosures were categorised into three groups: 'Nocturnal Houses', where kiwi were housed for public display on a reversed lighting schedule; 'Outside On Display', where kiwi were housed in natural outdoor enclosures but the public were encouraged to view kiwi at night

time; and ‘Off Display’. Off Display enclosures were anywhere the kiwi were not expected to be visible to the public. This includes enclosures where the public may walk past/through during the day, but kiwi are asleep in their burrows so are not expected to be on view. While kiwi could be seen if they were awake and walking about, facilities do not define these enclosures as on display.

Respondents were then asked if they had any Nocturnal Houses. If they responded yes, they were asked about variables assumed to be the same across all enclosures within the nocturnal house including questions about substrate (mc), enrichment and feeding procedures (multi-choice question (mc)), lighting colour (mc), air temperature and humidity levels and regulation (binary yes/no question (b), follow up asking for levels; o), barriers (mc), nest box access (b) and keeper presence (mc).

Next, questions were asked about each individual enclosure within a nocturnal house (to a maximum of six enclosures per house) including enclosure size (o) and number of nest boxes/dens (mc). Demographic information collected about kiwi living in each enclosure included age (o), weight (o), sex (b) and any past or present behavioural or health concerns (o). If a second kiwi shared the same enclosure (maximum of two birds per enclosure permitted; reference), these questions were repeated for that bird. If the facility had more than one nocturnal house, enclosure and demographic questions were repeated, to a maximum of three nocturnal houses.

Respondents were then asked about on-display outdoor enclosures. Data were collected on substrate, enrichment and feeding procedures, barriers, the number of kiwi in an enclosure, nest box access and keeper presence, and visitor access to the kiwi (o). The age, weight and any past or present behavioural or health concerns of kiwi living in these enclosures were also collected.

The maximum number of Nocturnal Houses, indoor enclosures per house and On Display enclosures included in the questionnaire were based on the initial discussions with the facilities.

The final questions concerned off-display enclosures and related to substrate, feeding and enrichment procedures. Wesley and Brader (2014) indicated the presence of kiwi indoor off-display enclosures internationally, so questions were asked about the location of each enclosure, including if visitors walked past the enclosure, or if the enclosure was behind the scenes and away from visitor areas. If the enclosure was indoors, questions were asked about lighting/temperature/humidity as for the nocturnal houses. As for other enclosure types, kiwi demographic data were also collected.

To reduce repetition, logic rules were applied to repeated questions where some consistency was expected. For example, it was expected that the substrate was sourced from the same location regardless of enclosure type or location. As such, a logic rule was applied so that the first given answer was provided in later questions with the response “Previous answer: xxx (please edit if incorrect)”.

The final question was open-ended asking “Are there any questions or concerns you have around the husbandry/welfare practices surrounding captive kiwi in NZ?”. The international and NZ questionnaires were identical except for the removal of ‘in NZ’ from this final question.
