# Supplementary material for: Survey of husbandry practices and captive environments for North Island brown kiwi (Apteryx mantelli) housed in facilities within and outside New Zealand
Source: Anim Welf. 2025 Jun 20;34:e41. doi: 10.1017/S0962728625100109 (PMC12284840; doi:10.1017/S0962728625100109)
Supplement: Connor et al. supplementary material 2 — Connor et al. supplementary material [file S0962728625100109sup002.pdf]

**Supplementary Material: Appendix B**
**Full Questionnaire**

## Kiwi Husbandry Survey

Thank you for participating in this research into the current husbandry practices for captive kiwi.

This survey is part of wider PhD research into the husbandry, behaviour and welfare of captive kiwi. The survey aims to gather information about current housing and management of kiwi in New Zealand and overseas facilities. The results will be used to focus future research efforts to support optimum captive management of kiwi and to better understand the influence of features of the captive environment and management procedures on kiwi health, behaviour and welfare.

The research is being conducted under the supervision of Nicholas Ling and Clare Browne at the University of Waikato, Ngaio Beausoleil at Massey University, New Zealand, and Kris Descovich at the University of Queensland, Australia, and with the approval of the Zoo and Aquarium Association (ZAA Manager, NZ Partnerships & Conservation, Todd Jenkinson).

This survey is not designed to critique the management of captive kiwi in any individual facility, but rather aims to develop an overview of actual practice and identify gaps in understanding about the potential effects of environmental features on kiwi behaviour and welfare so that they can be further studied. As a participant in this study, you and your facility will remain anonymous in any material produced from the research. The data collected will be used in a PhD dissertation, conference papers and journal articles. De-identified raw data from this study will be kept in secure storage for 5 years, as per University of Waikato policy, and any personal data will be destroyed at the completion of the project. Information identifying the institution and individual kiwi will be removed from data before publishing but may be used by the researchers to identify facilities and kiwi for possible further observational behaviour research. Information about individual kiwi will not be linked to specific facilities and results will be presented at the population level.

You will be asked about each of your enclosure types, and about your individual kiwi. Questions will cover substrate, lighting regime, feeding, enrichment, behavioural and health issues, and facility hours and visitor numbers. The length of the survey will depend on the number of different enclosure types, and the number of kiwi housed in your facility, taking an

average of ten minutes per enclosure.

By ticking the ‘participate’ box below you are indicating that you have read this information sheet and give your consent to take part. Participation in this survey is completely voluntary. There are no risks associated with completing the survey. As a participant, you have the right to ask any question about the study. You can leave the survey and return to it at any stage if you need time to gather information. You may decline to answer any question in the survey or terminate your participation in the survey at any point. If required, you may withdraw from the survey and withdraw all data contributed without penalty or repercussion at any point up to two weeks after our follow up call.

If you have any questions, please get in touch with Rebecca (Rebecca.L.Connor@gmail.com) or her supervisor Nicholas Ling (nicholas.ling@waikato.ac.nz).

This research project has been approved by the Human Research Ethics Committee of the University of Waikato under HREC(HECS)2021#53. For any ethical questions or concerns please contact the Chair of the Committee, email hecs-ethics@waikato.ac.nz, postal address, University of Waikato, Te Whare Wananga o Waikato, Private Bag 3105, Hamilton 3240.

○ I agree to participate in this research (1)

| Section | Q | Instructions/Question | Answer Type | Answer Options | Survey Logic |
|---|---|---|---|---|---|
| **Facility** | 1 | What is the name of your facility? | Open | | |
| | 2 | How many visitors (on average/approximately) attend your facility on weekend days? | Open | | |
| | 3 | How many visitors (on average/approximately) attend your facility on week days? | Open | | |
| | 4 | What time do visitors have access to your kiwi enclosures? (Opening until closing) | Open | | |
| **Diet** | 5 | What do you feed your kiwi? (e.g., the Massey University diet, or the Massey diet with adaptations such as extra vitamins, or something else?) | Open | | |
| | 6 | How is kiwi food presented to the kiwi? | Multi Choice | Test tubes<br>Bowl/dish<br>Other (Type) | |
| **Health Checks** | 7 | How often are health checks (by keepers) given to kiwi? What does this entail? (e.g., visual check, physical check, etc) | Form Field - Open | Daily (Type in)<br>Twice Weekly (Type in)<br>Weekly (Type in)<br>Fortnightly (Type in)<br>Monthly (Type in)<br>Bi-Monthly (Type in)<br>6 monthly (Type in)<br>Yearly (Type in)<br>Other (Type in) | |
| | 8 | Do you have a health check form or record that is used/kept by keepers? | Binary | Yes<br>No | |
| | 9 | How often are kiwi health checked by a vet? | Multi Choice | Monthly<br>Bi-Monthly<br>6 monthly<br>Yearly<br>Only if showing signs of sickness<br>Other (Type in) | |
| | 10 | How often are kiwi weighed? | Multi Choice | Monthly<br>Bi-Monthly<br>6 monthly<br>Yearly<br>Other (Type in) | |
| | 11 | What method/model is used to evaluate the body condition of your kiwi? | Open | | |
| **Data collection** | 12 | Do you routinely collect information about visitor behaviour to your kiwi enclosures? | Binary/Text | Yes (Please provide details of how you collect the data and what it is used for)<br>No | |
| | 13 | Do you routinely collect information about the behaviour of your kiwi? | Binary/Text | Yes (Please provide details of how you collect the data and what it is used for)<br>No | |
| | | **Nocturnal houses** | | | |
| **Nocturnal houses** | 14 | How many nocturnal houses do you have? (i.e., a room or building where kiwi are housed in indoor enclosures on a reverse lighting schedule for visitors to see kiwi. Please note, this question relates to the number of individual buildings holding kiwi, NOT the number of enclosures within the house) | Multi Choice | 0<br>1<br>2<br>3<br>Other (Type in) | Skip To: "Outside On-Display" (Q52) if 0 |
| **Flooring and leaf litter** | 15 | Is the substrate (flooring) made up of soil and leaf litter? | Binary/Text | Yes<br>No (please type in what is used for the substrate) | |

| Section | Q | Instructions/Question | Answer Type | Answer Options | Survey Logic |
|---|---|---|---|---|---|
| | 16 | How often is leaf litter added to enclosures (i.e., topped up) in nocturnal house? | Multi Choice | Daily<br>Twice Weekly<br>Weekly<br>Fortnightly<br>Monthly<br>Bi-Monthly<br>6 monthly<br>Yearly<br>Other (Type in) | |
| | 17 | Where is the leaf litter sourced from for nocturnal houses? | Open | | |
| | 18 | How is soil or leaf litter filtered or processed before being placed in the enclosures in the nocturnal houses? | Open | | |
| | 19 | How long between the leaf litter being collected and spread through out the enclosure in the nocturnal houses? | Multi Choice | Less than 12 hours<br>12 to 24 hours<br>24 to 48 hours<br>48 to 72 hours<br>72 hours plus<br>Other (Please type in) | |
| | 20 | How is soil or leaf litter filtered or processed before being placed in enclosure? | Open | | |
| | 21 | How often is the substrate (or part of) wet down in the nocturnal house? | Multi Choice | Never<br>Daily<br>Twice Weekly<br>Weekly<br>Fortnightly<br>Monthly<br>Bi-Monthly<br>6 monthly<br>Yearly<br>Other (Type in) | |
| | 22 | How often is the substrate (or part of) turned over in enclosures in the nocturnal house? | Multi Choice | Never<br>Daily<br>Twice Weekly<br>Weekly<br>Fortnightly<br>Monthly<br>Bi-Monthly<br>6 monthly<br>Yearly<br>Other (Type in) | |
| | 23 | How often are subsoil and leaf litter replaced completely in the nocturnal houses? | Multi Choice | 6 monthly<br>Yearly<br>Bi-Yearly<br>Other (Type in) | |
| | | | **Nocturnal House Enclosures** | | |
| **Nocturnal house X** | | **These questions refer to the enclosures within your x$^{st}$ Nocturnal house** | | | |
| **Lighting** | 24 | Does the light regime (amount of time/start time in night phase/light phase) change over the year or remain static? | Multi Choice | Static (no change)<br>Seasonal change<br>Other change (Open) | |
| | 25 | What is the start time of the following currently in the nocturnal house (approximately)? | Form Field - Open | Dusk' lighting (if applicable) (Please Type)<br>Night time lighting (Please Type)<br>'Dawn' lighting (if applicable (Please Type)<br>Day time lighting (Please Type) | |

| Section | Q | Instructions/Question | Answer Type | Answer Options | Survey Logic |
|---|---|---|---|---|---|
| | 26 | What colour of lighting is used in the nocturnal house? | Multi Choice | Red<br>Blue<br>Yellow/Orange<br>White<br>Mixed colour (Open)<br>Other (Open) | |
| | 27 | Is any UV lighting used in the nocturnal house enclosures? | Binary | Yes<br>No | |
| **Sound and Visitors** | 28 | Is a sound scape (I.e., wind sound, bird song) provided in the nocturnal house? | Binary/Text | Yes (please give details of what is used and how)<br>No | |
| | 29 | What sort of window/barrier exists between kiwi and visitors? | Open | Wooden Fence (please enter approx height)<br>Glass/wall barrier to visitor head height or below<br>Glass/wall barrier to visitor head height or above (not full barrier, does not reach roof)<br>Full barrier to ceiling with walls/single glazed glass<br>Full barrier to ceiling with walls/double glazed glass<br>Other (please type in) | |
| | 30 | Is Kiwi enclosure floor at visitor ground level or raised? | Binary/Text | Ground Level<br>Raised (please give details of how far the enclosure floor is raised off the visitor floor level) | |
| **Food and Enrichment** | 31 | What time/how often are kiwi in the Nocturnal house fed? | Open | | |
| | 32 | What sort of enrichment is provided for the kiwi? How often is it provided? (Please select all that apply) | Form Field - Open | Leaf litter (Please enter frequency of supply<br>Logs (Please enter frequency of supply<br>Worms (Please enter frequency of supply<br>Other Insects (Please enter frequency of supply<br>Other (Open) | |
| **Temperature and Humidity** | 33 | Is the temperature in the nocturnal house regulated? | Binary/Text | Yes (please type in set temp in degrees celsius)<br>No | |
| | 34 | Is the humidity in the nocturnal house regulated? | Binary/Text | Yes (please type the humidity level you attempt to maintain)<br>No | |
| **Data collection** | 35 | Do you have video cameras installed inside the enclosures of the nocturnal house? | Binary | Yes<br>No | |
| | 36 | Do you have video cameras installed inside the nest boxes/dens in the nocturnal house? | Binary | Yes<br>No | |
| | 37 | Do kiwi have access to nest boxes during their night period? | Binary | Yes<br>No<br>Other (type in) | |
| **Keepers** | 38 | How many keepers have contact with kiwi in this nocturnal house? | Open | | |
| | 39 | How often do keepers enter the enclosures during kiwi night time period? | Multi Choice | Once daily<br>Twice daily<br>Three times daily<br>Four times daily<br>Other (please type in) | |

| Section | Q | Instructions/Question | Answer Type | Answer Options | Survey Logic |
|---|---|---|---|---|---|
| | 40 | How often do keepers enter the enclosures during kiwi day time period? | Multi Choice | Once daily<br>Twice daily<br>Three times daily<br>Four times daily<br>Other (please type in) | |
| | | | **Nocturnal House Enclosure** | | |
| **Enclosure X** | | **These questions refer to the X enclosures within your X Nocturnal house** | | | |
| | 41 | What is the size of the enclosure (m2)? | Open | | |
| | 42 | How many nest boxes are in the enclosure? | Open | | |
| | 43 | What is the name of the 1st kiwi housed in this enclosure? | Open | | Provided name is used in kiwi questions |
| **Kiwi X** | 44 | How old is (kiwi name)? | Open | | |
| | 45 | What sex is (Kiwi name)? | Binary | Male<br>Female | |
| | 46 | How long has (Kiwi name) | Form Field - Open | Been in this enclosure? (Please type)<br>Been on display for the public (in any on display enclosure within your facility)? (Please type) | |
| | 47 | What is the most recent weight of (kiwi name)? | Open | | |
| | 48 | Do you (or other keepers) have any particular behavioural concerns regarding (name)? | Open | | |
| | 49 | Are there any particular health concerns regarding (Kiwi name)? Any medical history? | Open | | |
| | 50 | Is there a second kiwi housed in this enclosure? | Binary/Text | Yes (please type in name)<br>No | Yes - repeat kiwi questions (Q44-59)<br>No - go to Question 51 |
| | 51 | Is there a x+1 enclosure in this nocturnal house? | Binary | Yes<br>No | Yes - repeat enclosure questions (Q41-50)<br>No - go to outdoor on-display questions (Q52) |
| Nocturnal House questions 24-53 repeat based on answer to Q14, How many Nocturnal houses do you have? | | | | | |
| **Outdoor on-display enclosures** | | | | | |
| | **52** | Do you have any outdoor on-display enclosures (where visitors can see kiwi in outdoor enclosures after dark)? | Binary | Yes<br>No | Yes - continue<br>No - go to Off-display questions (Q83) |
| | **53** | What time do visitors have access to the outdoor on display enclosures? (Opening of display until closing) | Open | | |
| | **54** | What sort of window/barrier exists between kiwi and visitors? | Multi Choice | Barrier to adult waist height or below<br>Barrier between adult waist height and adult height height<br>Barrier between adult head height and ceiling (not full barrier, does not reach roof)<br>Full barrier to ceiling with walls/single glazed glass<br>Full barrier to ceiling with walls/double glazed glass<br>Other (please type in) | |
| | **55** | Is Kiwi enclosure floor at visitor ground level or raised? | Binary/Text | Ground Level<br>Raised (please give details of how far the enclosure floor is raised off the visitor floor level) | |
| **Flooring and leaf litter** | **56** | Is the substrate (flooring) made up of soil and leaf litter? | Binary/Text | Yes<br>No (please type in what is used for the substrate) | |

| Section | Q | Instructions/Question | Answer Type | Answer Options | Survey Logic |
|---|---|---|---|---|---|
| | **57** | How often is leaf litter added to enclosures (i.e., topped up)? | Multi Choice | Daily<br>Twice Weekly<br>Weekly<br>Fortnightly<br>Monthly<br>Bi-Monthly<br>6 monthly<br>Yearly<br>Other (Type in) | |
| | **58** | Where is leaf litter sourced from? | Open | Previous answer: {X} (please edit if incorrect). | Provides previous answer from Noc house questions |
| | **59** | How long  between the leaf litter being collected and spread through out the enclosure? | Multi Choice | Less than 12 hours<br>12 to 24 hours<br>24 to 48 hours<br>48 to 72 hours<br>72 hours plus<br>Other (Please type in) | |
| | **60** | How is soil or leaf litter filtered or processed before being placed in the enclosures? | Open | Previous answer: {X} (please edit if incorrect). | Provides previous answer from Noc house questions |
| | **61** | How often is the substrate (or part of) wet down? | Multi Choice | Never<br>Daily<br>Twice Weekly<br>Weekly<br>Fortnightly<br>Monthly<br>Bi-Monthly<br>6 monthly<br>Yearly<br>Other (Type in) | |
| | **62** | How often is the substrate (or part of) turned over in enclosures? | Multi Choice | Never<br>Daily<br>Twice Weekly<br>Weekly<br>Fortnightly<br>Monthly<br>Bi-Monthly<br>6 monthly<br>Yearly<br>Other (Type in) | |
| | **63** | How often are subsoil and leaf litter replaced completely in the nocturnal houses? | Multi Choice | 6 monthly<br>Yearly<br>Bi-Yearly<br>Other (Type in) | |
| **Food and Enrichment** | **64** | What time/how often are kiwi fed? | Open | | |
| | **65** | What sort of enrichment is provided for kiwi? | Form Field - Open | Leaf litter (Please enter frequency of supply<br>Logs (Please enter frequency of supply<br>Worms (Please enter frequency of supply<br>Other Insects (Please enter frequency of supply<br>Other (Open) | |
| **Data collection** | **66** | Do you have video cameras installed inside the enclosures of the outside on display enclosures? | Binary | Yes<br>No | |

| Section | Q | Instructions/Question | Answer Type | Answer Options | Survey Logic |
|---|---|---|---|---|---|
| | **67** | Do you have video cameras installed inside the nest boxes/dens in the outside on display enclosures? | Binary | Yes<br>No | |
| | **68** | Do kiwi have access to nest boxes during their night period? | Binary | Yes<br>No<br>Other (type in) | |
| **Keeper** | **69** | How many keepers have contact with kiwi in this outside on display enclosures? | Open | Previous answer: {X} (please edit if incorrect). | Provides previous answer from Noc house questions |
| | **70** | How often do keepers enter the enclosures during kiwi night time period? | Multi Choice | Once daily<br>Twice daily<br>Three times daily<br>Four times daily<br>Other (please type in) | |
| | **71** | How often do keepers enter the enclosures during kiwi day time period? | Multi Choice | Once daily<br>Twice daily<br>Three times daily<br>Four times daily<br>Other (please type in) | |
| | | **Outdoor On Display Enclosure** | | | |
| **Enclosure X** | | **Outdoor on display enclosure**<br>**These questions refer to the X outside on display enclosure.** | | | Repeat Q72-82 for every outdoor on-display enclosure |
| | **72** | What is the approximate size of the enclosure (m2)? | Open | | |
| | **73** | How many nest boxes are in the enclosure? | Multi Choice | 0<br>1<br>2<br>Other (type in) | |
| **Kiwi X** | **74** | What is the name of the 1st kiwi housed in this enclosure? | Open | | Name is used in kiwi questions |
| | **75** | How old is (kiwi name)? | Open | | |
| | **76** | What sex is (Kiwi name)? | Binary | Male<br>Female | |
| | **77** | How long has (Kiwi name) | Form Field - Open | Been in this enclosure? (Please type)<br>Been on display for the public (in any on display enclosure within your facility)? (Please type) | |
| | **78** | What is the most recent weight of (kiwi name)? | Open | | |
| | **79** | Do you (or other keepers) have any particular behavioural concerns regarding (name)? | Open | | |
| | **80** | Are there any particular health concerns regarding (Kiwi name)? Any medical history? | Open | | |
| | **81** | Is there a second kiwi housed in this enclosure? | Binary and Text | Yes (please type in name)<br>No | Yes - repeat kiwi questions (Q75-80)<br>No - go to question 82 |
| **Enclosure X+1** | **82** | Do you have a x+1 Outside On display enclosure? | Binary | Yes<br>No | Yes - repeat enclosure questions (Q72-81)<br>No - go to question 83 |
| **Off-Display enclosures** | | | | | |
| | **83** | Do you have enclosures where birds are held for off-display purposes? | Binary | Yes<br>No | Yes - continue<br>No - go to question 118 |
| **Flooring and leaf litter** | **84** | Is the substrate (flooring) made up of soil and leaf litter? | Binary and Text | Yes<br>No (please type in what is used for the substrate) | |

| Section | Q | Instructions/Question | Answer Type | Answer Options | Survey Logic |
|---|---|---|---|---|---|
| | 85 | How often is leaf litter added to enclosures (i.e., topped up)? | Multi Choice | Daily<br>Twice Weekly<br>Weekly<br>Fortnightly<br>Monthly<br>Bi-Monthly<br>6 monthly<br>Yearly<br>Other (Type in) | |
| | 86 | Where is the leaf litter sourced from? | | Previous answer: {X} (please edit if incorrect). | Provides previous answer from Noc house questions |
| | 87 | How long  between the leaf litter being collected and spread through out the enclosure? | Multi Choice | Less than 12 hours<br>12 to 24 hours<br>24 to 48 hours<br>48 to 72 hours<br>72 hours plus<br>Other (Please type in) | |
| | 88 | How is soil or leaf litter filtered or processed before being placed in enclosure? | Open | Previous answer: {X} (please edit if incorrect). | Provides previous answer from Noc house questions |
| | 89 | How often is the substrate (or part of) wet down? | Multi Choice | Never<br>Daily<br>Twice Weekly<br>Weekly<br>Fortnightly<br>Monthly<br>Bi-Monthly<br>6 monthly<br>Yearly<br>Other (Type in) | |
| | 90 | How often is the substrate (or part of) turned over? | Multi Choice | Never<br>Daily<br>Twice Weekly<br>Weekly<br>Fortnightly<br>Monthly<br>Bi-Monthly<br>6 monthly<br>Yearly<br>Other (Type in) | |
| | 91 | How often are subsoil and leaf litter replaced completely? | Multi Choice | 6 monthly<br>Yearly<br>Bi-Yearly<br>Other (Type in) | |
| **Food and Enrichment** | 92 | What time/how often are kiwi fed? | Open | | |
| | 93 | What sort of enrichment is provided for kiwi? | Form Field - Open | Leaf litter (Please enter frequency of supply<br>Logs (Please enter frequency of supply<br>Worms (Please enter frequency of supply<br>Other Insects (Please enter frequency of supply<br>Other (Open) | |
| **Data collection** | 94 | Do you have video cameras installed inside the enclosures? | Binary | Yes<br>No | |

| Section | Q | Instructions/Question | Answer Type | Answer Options | Survey Logic |
| --- | --- | --- | --- | --- | --- |
| | 95 | Do you have video cameras installed inside the nest boxes/dens? | Binary | Yes<br>No | |
| | 96 | Do kiwi have access to nest boxes during their night period? | Binary | Yes<br>No<br>Other (type in) | |
| **Keeper** | 97 | How many keepers have contact with kiwi in this nocturnal house? | Open | Previous answer: {X} (please edit if incorrect). | Provides previous answer from Noc house questions |
| | 98 | How often do keepers enter the enclosures during kiwi night time period? | Multi Choice | Once daily<br>Twice daily<br>Three times daily<br>Four times daily<br>Other (please type in) | |
| | 99 | How often do keepers enter the enclosures during kiwi day time period? | Multi Choice | Once daily<br>Twice daily<br>Three times daily<br>Four times daily<br>Other (please type in) | |
| **Off Display Enclosure** | | | | | |
| **Enclosure X** | | **Off Display enclosure**<br>**These questions refer to the X off display enclosure.** | | | |
| | 100 | Where is the enclosure based? | Multi Choice | Outside in an area visitors have no access to<br>Outside in an area visitors walk past<br>Inside<br>Other (Please type in) | |
| | 101 | What fencing is separating the enclosure from visitors? | Multi Choice | Fence to visitor waist height or lower<br>Fence from visitor waist to head height<br>Full fence (above 6 foot)<br>Other (please type in) | Displays if "Outside in an area visitors walk past" is selected in Q100 |
| **Enclosure Lighting** | 102 | Is the lighting on natural or reverse timing? | Binary | Natural<br>Reverse<br>Other (please type in) | Enclosure lighting questions (Q102-106) display if "Inside" is selected in Q100 |
| | 103 | Does the light regime (amount of time/start time in night phase/light phase) change over the year or remain static? | Multi Choice | Static (no change)<br>Seasonal change<br>Other change (Open) | |
| | 104 | What is the start time of the following currently? | Form Field - Open | Dusk' lighting (if applicable) (Please Type)<br>Night time lighting (Please Type)<br>'Dawn' lighting (if applicable (Please Type)<br>Day time lighting (Please Type) | |
| | 105 | What colour of lighting is used? | Multi Choice | Red<br>Blue<br>Yellow/Orange<br>White<br>Mixed colour (Please type)<br>Other (Please type) | |
| | 106 | Is any UV lighting used in the enclosure? | Binary | Yes<br>No | |
| **Enclosure X** | 107 | What is the approximate size of the enclosure (m2)? | Open | | |
| | 108 | How many nest boxes are in the enclosure? | Multi Choice | 0<br>1<br>2<br>Other (type in) | |
| | 109 | What is the name of the 1st kiwi housed in this enclosure? | Open | | Name is used in kiwi questions |

| Section | Q | Instructions/Question | Answer Type | Answer Options | Survey Logic |
|---|---|---|---|---|---|
| **Kiwi X** | **110** | How old is (kiwi name)? | Open | | |
| | **111** | What sex is (Kiwi name)? | Binary | Male<br>Female | |
| | **112** | How long has (Kiwi name) | Form Field - Open | Been in this enclosure? (Please type)<br>Been on display for the public (in any on display enclosure within your facility)? (Please type) | |
| | **113** | What is the most recent weight of (kiwi name)? | Open | | |
| | **114** | Do you (or other keepers) have any particular behavioural concerns regarding (name)? | Open | | |
| | **115** | Are there any particular health concerns regarding (Kiwi name)? Any medical history? | Open | | |
| | **116** | Is there a second kiwi housed in this enclosure? | Binary/Text | Yes (please type in name)<br>No | Yes - repeat kiwi questions (Q110-115)<br>No - go to question 117 |
| **Enclosure X+1** | **117** | Do you have a x+1 off display enclosure? | Binary | Yes<br>No | Yes - repeat enclosure questions (Q100-116)<br>No - go to Q118 |
| **Final Question** | **118** | Are there any questions or concerns you have around the husbandry/welfare practices surrounding captive Kiwi? This could be issues with individual kiwi (response to particular noises) your facility (particular visitor behaviour, husbandry routines) or in general. | Open | | |
| | | | | | |
| | | | | | |
| | | | | | |
| | | | | | |
| | | | | | |
| | | | | | |
| | | | | | |
